# Targeting Dysregulation of Metalloproteinase Activity in Osteoarthritis

**DOI:** 10.1007/s00223-020-00739-7

**Published:** 2020-08-09

**Authors:** Kazuhiro Yamamoto, David Wilkinson, George Bou-Gharios

**Affiliations:** Institute of Life Course and Medical Sciences, University of Liverpool, William Duncan Building, 6 West Derby Street, Liverpool, L7 8TX UK

**Keywords:** Extracellular matrix, Cartilage, Endocytosis, Metalloproteinase, TIMP, Sulphated glycosaminoglycan, Serine proteinase

## Abstract

Metalloproteinases were first identified as collagen cleaving enzymes and are now appreciated to play important roles in a wide variety of biological processes. The aberrant activity and dysregulation of the metalloproteinase family are linked to numerous diseases including cardiovascular and pulmonary diseases, chronic wounds, cancer, fibrosis and arthritis. Osteoarthritis (OA) is the most prevalent age-related joint disorder that causes pain and disability, but there are no disease-modifying drugs available. The hallmark of OA is loss of articular cartilage and elevated activities of matrix-degrading metalloproteinases are responsible. These enzymes do not exist in isolation and their activity is tightly regulated by a number of processes, such as transcription, proteolytic activation, interaction with their inhibitors, cell surface and extracellular matrix molecules, and endocytic clearance from the extracellular milieu. Here, we describe the functions and roles of metalloproteinase family in OA pathogenesis. We highlight recent studies that have illustrated novel mechanisms regulating their extracellular activity and impairment of such regulations that lead to the development of OA. We also discuss how to stop or slow down the degenerative processes by targeting aberrant metalloproteinase activity, which may in future become therapeutic interventions for the disease.

## Introduction

Matrix metalloproteinases (MMPs), adamalysins (or a disintegrin and metalloproteinases (ADAMs)), and ADAM with thrombospondin motifs (ADAMTSs) belong to the family of zinc endopeptidases collectively referred to as metzincins. The metzincin superfamily is distinguished by a highly conserved motif containing three histidines that bind to zinc at the catalytic site and a conserved methionine that sits beneath the active site [1].

The human genome contains 23 MMP genes and most MMPs have several domains: a signal peptide, a propeptide, a catalytic metalloproteinase domain, a linker (hinge) peptide and a hemopexin domain [2]. The family includes collagenases, gelatinases, stromelysins, matrilysins, membrane-type MMPs and others. MMPs were initially characterised as extracellular matrix (ECM)-degrading proteinases. In 1962, Gross and Lapiere were the first to describe the activity of a collagenase in the resorbing tadpole tail during metamorphosis [3]. It is now considered that their actions go far beyond ECM proteolysis, and influence cell behaviour by releasing growth factors, generating neo-ligands from carrier proteins and cell surface molecules, inactivating proteinase inhibitors, and modulating inflammatory mediators [4]. MMPs thus have important roles in development, morphogenesis, tissue remodelling, tissue repair, angiogenesis, inflammation, and innate immunity.

The ADAM family is conserved type I transmembrane metalloproteinases related to the MMPs and ADAMTSs with human genome containing 20 ADAMs. The proteolytically active ADAMs mainly function as ‘sheddases’, cleaving the juxta-membrane region of their trans-membrane substrates to release the soluble ectodomain of the substrate to the extracellular milieu [5]. This activity enables them to regulate the extracellular availability of autocrine and paracrine signalling molecules, such as transmembrane cytokines and growth factors, and their receptors.

ADAMTS proteinases degrade ECM components and are secreted multidomain metalloproteinases, consisting of a signal peptide, a pro-domain, a metalloproteinase domain, a disintegrin domain, a thrombospondin type I motif, a cysteine-rich domain, a spacer domain, and a second thrombospondin motif of variable numbers of repeats at the C-terminal region [6]. The human genome contains 19 ADAMTS proteinases and their evolutionary conservation and expansion in mammals is suggestive of crucial embryologic or physiological roles in humans. Mendelian disorders or birth defects resulting from naturally occurring ADAMTS-2, 3, 10, 13, 17, 20 mutations as well as numerous phenotypes identified in genetically engineered mice further indicate ADAMTS participation in major biological pathways [7].

Most, if not all, of the metalloproteinases are tightly regulated by a number of ways, such as transcriptional regulation, proteolytic activation and interaction with tissue inhibitors of metalloproteinases (TIMPs). Not surprisingly, aberrant activity due to dysregulation of these metalloproteinase family are linked to numerous diseases including cardiovascular diseases, chronic wounds, pulmonary diseases, cancer, fibrosis, rheumatoid arthritis (RA), and osteoarthritis (OA) [8, 9].

OA is the most prevalent age-related joint disorder that causes pain and disability, but there is no disease-modifying intervention currently available, except surgery at end stage of the disease. The hallmark of OA is manifested by progressive degradation of articular cartilage ECM due to elevated activities of matrix-degrading metalloproteinases. In this review, we describe the functions and roles of metalloproteinase family in articular cartilage integrity and degradation. We highlight recent studies that have illustrated novel mechanisms regulating the extracellular activity of cartilage-degrading metalloproteinases and their inhibitors. We also discuss how to stop or slow down the degenerative processes in OA by targeting impairment of such regulations, which may become therapeutic interventions for the disease.

## Metalloproteinases in OA Pathogenesis

### Aggrecanases, a Group of ADAMTSs, Play a Major Role in Aggrecan Degradation in Cartilage

Degradation of aggrecan, the major proteoglycan in articular cartilage is an early event in the pathophysiology of OA and a considerable amount of research has been carried out to identify the enzyme(s) responsible. MMP-3 was isolated from human articular cartilage [10] and found to cleave the Asn^341^ ~ Phe^342^ bond (where ~ indicates the cleavage site) in the aggrecan inter-globular domain (IGD) [11]. Several other MMPs, including MMP-1, 2, 7, 8, 9 and 13, were later found to be able to cleave the same site, as well as other sites towards the C-terminus of the molecule [12]. However, the contribution of MMP-mediated aggrecan cleavage to the OA pathology is under debate [13]. Sandy et al.[14] revealed that the majority of aggrecan fragments present in the synovial fluid of OA patients were cleaved not at the MMP-sensitive Asn^341^ ~ Phe^342^ bond, but at the Glu^373^ ~ Ala^374^ bond in the IGD.

Aggrecanase activity was first defined as the ability to cleave at the Glu^373^ ~ Ala^374^ bond in the IGD and this cleavage causes aggrecan depletion from cartilage and ablation of the molecule’s function in cartilage. The first aggrecanase was identified as a member of the ADAMTS family and designated as ADAMTS-4 (aggrecanase 1) [15]). ADAMTS-1, 5 (aggrecanase 2), 8, 9, 15, 16 and 18 also have aggrecanase activity, but among these, ADAMTS-5 is the primary aggrecanase in mice. This was demonstrated by the finding that *Adamts5*^−/−^ mice develop less severe cartilage damage in a murine surgical model of OA and in an antigen-induced arthritis model, respectively [16, 17]. Similarly, transgenic mice with a knock-in mutation of aggrecan preventing ‘aggrecanase’ cleavage of the Glu^373^ ~ Ala^374^ bond also develop less severe OA in the surgical OA and antigen-induced arthritis models [18]. *Adamts1*^−/−^ and *Adamts4*^−/−^ mice are not similarly protected [19, 20]. ADAMTS-5 is approximately 30-fold more potent than ADAMTS-4 [21], supporting the view that ADAMTS-5 is the major aggrecanase in cartilage catabolism. While there is some evidence that ADAMTS-4 may contribute to cartilage degradation in humans [22, 23], recent studies by Larkin et al.[24] with neutralizing monoclonal antibodies have shown that ADAMTS-5 is more effective than ADAMTS-4 in aggrecan degradation in human OA cartilage and non-human primates in vivo. Furthermore, a recent study revealed that ADAMTS-5 also cleaves inter-α-inhibitor and releases active heavy chain 2, which is detectable in synovial fluids from both RA and OA patients, and may contribute to the progression of arthritis beyond the degradation of aggrecan [25].

### Collagen Fibrils are Degraded by Collagenases, a Group of MMPs

Whilst aggrecan loss can be reversed, collagen degradation is irreversible, and cartilage cannot be repaired once collagen is destroyed [26, 27]. Type II collagen is extremely resistant to degradation by most proteinases because of its triple-helical structure. Only the classical collagenases including MMP-1, 8, and 13, and to a much lesser extent, MMP-14, are able to degrade triple-helical type II collagen fibrils into three-quarter and one-quarter fragments [28]. This is a crucial step for collagenolysis in the tissue, and denaturing them into gelatin, which can then be subsequently digested into small peptides by the gelatinases (MMP-2 and 9). The exact order in which cartilage matrix components are degraded during the development of OA is difficult to ascertain, but a number of in vitro studies on cartilage explants suggest that collagen degradation occurs only after aggrecan is lost from the tissue, and that the presence of aggrecan protects the collagen from degradation [29, 30].

The first human collagenase to be purified was MMP-1 (collagenase-1), isolated from rheumatoid synovium [31], where it was localized in the synovial lining cells at the junction of pannus and cartilage [32], suggesting its role in tissue destruction. MMP-1 also efficiently cleaves type II collagen (R. Visse, Y. Tominaga, M. Wang, H. Nagase, personal communication), but its role in OA cannot be studied using murine models as murine MMP-1 differs considerably from the human enzyme [33]. MMP-8 is mainly produced by neutrophils, although it is also expressed by a wide range of cells including chondrocytes [34] and synovial fibroblasts [35]. A role of MMP-8 in RA pathogenesis has been suggested [36, 37] but little is known about its role in OA. MMP-13 (collagenase-3) is considered as a major collagenase in the development of OA [38] because of its elevated expression in human OA cartilage and its effective ability to degrade collagen II fibrils [39–41]. Further support for this is a study with *Mmp13*^−/−^ mice, whose cartilage was protected from degradation in the surgically induced OA model [42]. In addition to fibrillar collagen types I, II and III, MMP-13 cleaves other ECM molecules such as N-terminal non-helical telopeptides of type I collagen, gelatins, type IV, IX, X, and XIV collagens, large tenascin C, fibronectin, aggrecan, perlecan, fibrillin-1, and osteonectin [43]. Another collagenase, MMP-14, is a membrane-bound protein and has been shown to promote invasion of rheumatoid synovial fibroblasts into cartilage [44]. It is highly expressed in rheumatoid synovial lining cells but similarly expressed in normal and OA cartilage [45–47]. The role of MMP-14 in OA has not been studied in murine surgical models as *Mmp14*^−/−^ mice exhibit severe skeletal abnormalities that lead to early death [48].

### MMP-13 in Bone ECM Remodelling and Osteophyte Formation

OA is now widely accepted as a whole joint disease [49]. In addition to the cartilage degradation, synovitis (synovial inflammation) and meniscal damage are common and altered levels of inflammatory mediators are detected in OA synovial fluid. Furthermore, osteophytes form in joint margins, and bone remodelling occurs, leading to bone marrow lesions and bone sclerosis [50]. The cross-talk between cartilage and other tissues suggests cartilage loss can occur secondary to above mentioned OA-related changes to the joint [51].

Osteocytes play an active role in remodelling their surrounding bone matrix—a process called perilacunar/canalicular remodelling (PLR) [52]. It is a dynamic process by which osteocytes secrete MMPs, cathepsin K and other enzymes to dynamically resorb and then replace the local bone matrix. Recently, Mazur et al.[53] established a novel mouse model in which MMP-13 is ablated in osteocytes, but not chondrocytes. They found that osteocyte-intrinsic deficiency in MMP-13 is sufficient to suppress PLR and induce premature OA accompanied by subchondral sclerosis and cartilage degradation in otherwise healthy young mice. This study highlights a new, causal role for osteocytic MMP-13 in the regulation of bone and cartilage homeostasis, and suggests reduction of PLR as a novel mechanism in OA.

## Extracellular Regulation of Cartilage-Degrading Proteinases

**Fig. 1:**
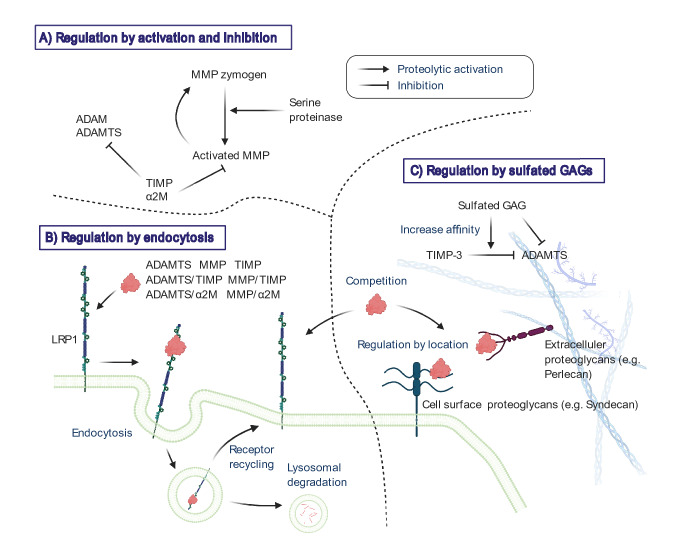
Extracellular regulation of cartilage-degrading metalloproteinase activities. Metalloproteinases are regulated in the extracellular environment by a number of mechanisms, including proteolytic activation of zymogens (**a**), interaction with endogenous inhibitors (TIMPs and α2M)(A), endocytic clearance mediated by cell surface scavenging receptor LRP1 (**b**), binding to the cell surface molecules and ECM via sulphated GAGs (**c**). Sulphated GAGs inhibit ADAMTSs activity by two modes of action including interacting with their ancillary domains and increasing TIMP-3 affinity for ADAMTSs (**c**). LRP1 and sulphated GAGs can compete each other for binding to several metalloproteinases and TIMP-3 (**b** and **c**)

The activity of metalloproteinases is tightly regulated by a number of mechanisms [54], and their activity is often not readily detected in steady state tissues (Fig. 1). Many of the MMPs are secreted from the cell in a zymogen form and are then activated extracellularly by other proteinases. The membrane-type MMPs (MMP-14, 15, 16, 17 and 24) and ADAMTSs are activated intracellularly by pro-protein convertases such as furin. The activity of mature metalloproteinases is regulated by endogenous inhibitors such as α2-macroglobulin (α2M) in blood plasma or body fluids and TIMPs in the tissue [55]. In addition to these regulations, cartilage-degrading metalloproteinases and their inhibitor TIMP-3 have been found to be very short-lived in the extracellular space, as they are rapidly endocytosed by the cells that produce them [56–60]. They also bind to sulphated glycosaminoglycans (GAGs) on the cell surface or in the ECM, with their extracellular availability determined by their relative affinity for each. These findings add further complexity to the regulation of activity of cartilage-degrading proteinases.

### Proteolytic Activation of Pro-MMPs by Serine Proteinases

The majority of MMPs are synthesised as zymogens and serine proteinases are likely physiological activators of pro-MMPs [61]. There are 178 serine proteinases in humans, making up approximately 1/3rd of the ‘degradome’ [62]. Serine proteinases harbour a ‘catalytic triad’ of serine^195^, Histidine^57^ and Aspartate^102^ (chymotrypsin nomenclature), which provide a charge relay system, allowing potent nucleophilic attack of substrate carbonyl bonds [63]. Serine proteinases can be broadly separated into trypsin-like, chymotrypsin-like and elastase-like proteinases, with particular residues in and surrounding the active site pockets, governing substrate specificity. Activation of pro-MMPs by serine proteinases usually occurs via an initial cleavage, which leads to the formation of a partially active intermediate enzyme, capable of the final processing into the fully active form [64, 65].

A well-established activator of MMPs is plasmin, an important serine proteinase in the fibrinolytic system. Plasmin is a broad-spectrum proteinase which is able to directly degrade many ECM components but also functions as a pro-MMP activator,  promoting ECM remodelling. These include MMP-1, 3, 13 and 14 [66]. Plasminogen mRNA expression is not detectable in human cartilage, but protein levels in the synovial fluid have been examined previously [67]. When plasminogen is added to cytokine-stimulated bovine cartilage, it promotes cartilage collagen release, suggesting the presence of plasminogen activators within this system [68].

In OA, cartilage collagen destruction can be initiated in the chondrocyte pericellular space [69], making membrane-bound serine proteinases of particular interest. The type II transmembrane serine proteinase (TTSP) matriptase is upregulated in OA cartilage and can activate both MMP-1 and 3. MMP-3 is well-known activator of other MMPs, including MMP-13 [70]. Addition of matriptase to human OA cartilage induces destruction of the cartilage ECM in an MMP-dependent manner [65, 71]. Matriptase can also activate proteinase-activated receptor 2 (PAR2), which induces the expression of MMPs in chondrocytes [65, 72]. Hepsin is a different TTSP which is also capable of activating MMP-1 and 3 but has a weaker propensity to cleave and activate PAR2 [73]. Importantly, TTSPs are able to auto-activate, a characteristic which has led to suggestions they lie at the top of proteolytic cascades.

### Inhibition by TIMPs

TIMPs are the endogenous inhibitors of the MMPs and some members of the ADAM and ADAMTS families [55]. There are four TIMPs expressed in humans and all MMPs tested to date are strongly inhibited by all four of the mammalian TIMPs (TIMP-1, 2, 3 and 4), with the exception of some of MT-MMPs that are poorly inhibited by TIMP-1. Besides MMPs, TIMP-1 inhibits ADAM10 and TIMP-3 inhibits ADAM10, 12, 17, 28 and 33, and ADAMTS-1, 2, 4 and 5 [55]. As TIMP-3 can inhibit both collagenases and aggrecanases, it is a central inhibitor for regulation of cartilage ECM turnover. Addition of exogenous TIMP-3, but not TIMP-1 or TIMP-2 blocks cartilage degradation in explant cultures [74], and injection of TIMP-3 blocks cartilage breakdown in a rat surgical model of OA [75]. The chondroprotective role of TIMP-3 is confirmed by the finding that *Timp3*^−/−^ mice develop increased cartilage degradation upon ageing [76] and increased cartilage damage in an antigen-induced arthritis model [77]. The susceptibility of other *Timp*-null mice to developing OA has not been reported, but the deterioration of bone quality in these mice render them ineffective model [78, 79] similar to the recent quadruble knock out of TIMPs [80]. TIMP-2 has little effect on aggrecan degradation in bovine, porcine or human cartilage explants, whilst TIMP-1 has been shown to partially inhibit aggrecan degradation in human but not bovine or porcine cartilage [30, 74, 81]. TIMP-3 mRNA levels are not significantly altered in OA [82–84], whereas expression of TIMP-4 is decreased in OA cartilage [46], and a single nucleotide polymorphism in the 3′ untranslated region of TIMP-4 is reportedly associated with OA in a Korean cohort [85].

### Endocytic Clearance by Cell Surface Receptor LRP1

Low-density lipoprotein (LDL) receptor-related protein 1 (LRP1 or CD91) is a member of a family of scavenger receptors related to the LDL receptor [86]. It is a type I transmembrane protein consisting of a 515-kDa α-chain containing the extracellular ligand-binding domains and a non-covalently associated 85-kDa β-chain containing a transmembrane domain and a short cytoplasmic tail. LRP1 was first characterized as a receptor for apolipoprotein E-containing lipoprotein particles [87] and for α2M-proteinase complexes [88]. This endocytic process is a general mechanism to eliminate excess active proteinases from tissues and body fluids, since most extracellular endopeptidases with different catalytic mechanisms can react with and be entrapped by α2M [89]. To date, more than 80 of structurally and functionally different molecules have been identified as LRP1 ligands [90, 91] and the list is still growing. The importance of LRP1 in biological processes is demonstrated by the lethality of LRP1 gene deletion at an early stage of murine embryonic development [92]. This indicates that endocytic scavenging of bioactive molecules is essential to maintain tissue homeostasis and that disruption of this process may result in pathological conditions.

The Partridge group first reported that rat MMP-13 disappeared from the culture medium of a rat osteoblast cell line and demonstrated that this occurred through a receptor-mediated process [93]. Subsequently, the disappearance was shown to be due to endocytosis of MMP-13 by LRP1 with the assistance of a 170 kDa MMP-13-specific receptor [94]. Yamamoto et al.[59] demonstrated that MMP-13 directly binds to cell surface LRP1 in human chondrocytes isolated from healthy adults, and it was constitutively expressed and secreted but rapidly endocytosed by the cells. Furthermore, both ADAMTS-4 and 5 also bind to LRP1 and their extracellular activity is tightly regulated by LRP1-mediated endocytic clearance in human cartilage [57, 58]. This regulation also applies to TIMP-3 [95, 96]. These findings suggest that cartilage-degrading proteinases and their inhibitors probably function for a very short period of time to maintain normal homeostatic turnover of ECM components of the tissue.

### Increase in Ectodomain Shedding of LRP1 in OA Cartilage

While ADAMTS-5 is considered as a major aggrecanase, its mRNA levels are not significantly elevated in human OA compared to normal cartilage [97, 98]. Studies by Yamamoto et al.[57] shed new insight into the regulation of ADAMTS-5 in OA, showing that endocytic clearance of ADAMTS-5 by LRP1 is impaired in OA chondrocytes. A reduction in MMP-13 endocytosis in OA chondrocytes was also reported [99]. In human OA cartilage, proteolytic shedding of LRP1 ectodomain is increased and two membrane-bound metalloproteinases MMP-14 and ADAM17 are the responsible sheddases [45]. Shed LRP1 retains ligand-binding capacity and can act as a decoy receptor [100]. Scilabra et al.[96, 101] reported that shed LRP1 competes with cell surface LRP1 for binding to TIMP-3, and that extracellular LRP1-TIMP-3 complexes retain their ability to inhibit target metalloproteinases. Shed LRP1 also binds to ADAMTS-4 and 5, and MMP-13, and prevents them being endocytosed without interfering with their activities [45]. Recently, Coveney et al.[102] demonstrated that disruption of intraflagellar transport protein 88, a core ciliary trafficking protein, increases LRP1 shedding and reduces endocytic clearance of ADAMTS-5 and MMP-13. As ectodomain cleavage of membrane protein occurs primarily on the cell surface, the ciliary machinery may regulate cell surface localisation of LRP1 and the sheddase enzymes.

### Regulation by Sulphated Glycosaminoglycans (GAGs)

Many metalloproteinases interact with heparan sulphate or chondroitin sulphate GAG chains of proteoglycans on the cell surface or in the ECM [103]. Binding of metalloproteinases to the cell surface could position the enzyme for directed proteolytic attack for activation of or by other proteinases and for regulation of other cell surface proteins. On the other hand, binding to the ECM could prevent loss of secreted enzyme, provide a reservoir of latent enzyme, and facilitate cellular sensing and regulation of enzyme levels [104]. In addition to these regulations by location, interaction of the metalloproteinases with sulphated GAGs can regulate their activity by dictating or limiting access to substrates, or by directly modulating activity [56].

Heparin, which is a highly sulphated GAG, binds to MMP-13 in vitro [105] and solubilises the enzyme from tissues [106]. Both ADAMTS-4 and 5 bind to the ECM via their non-catalytic C-terminal (ancillary) domains [21, 107] and heparin solubilises ADAMTS-5 from the ECM [21]. Nagase’s group found that heparin and calcium pentosan polysulphate, a chemically sulphated xylanopyranose, inhibit ADAMTS-4 and 5 by interacting with their ancillary domains [60, 108]. Sulphated GAGs also affect the interaction of metalloproteinases with TIMP-3, the only TIMP that binds to the ECM. It increases TIMP-3 affinity for ADAMTS-4 and 5 [60] and the ability of sulphated GAGs to increase their affinity is highly dependent on the sulphation pattern of the GAG [109].

Several of the metalloproteinases and TIMPs studied to date are able to bind to both LRP1 and sulphated GAGs, and they can compete with each other for binding to ligands [56]. This competition thus also regulates extracellular availability of cartilage-degrading proteinases and TIMP-3.

## Therapeutic Potential of Targeting Aberrant Metalloproteinase Activity in OA

**Fig. 2:**
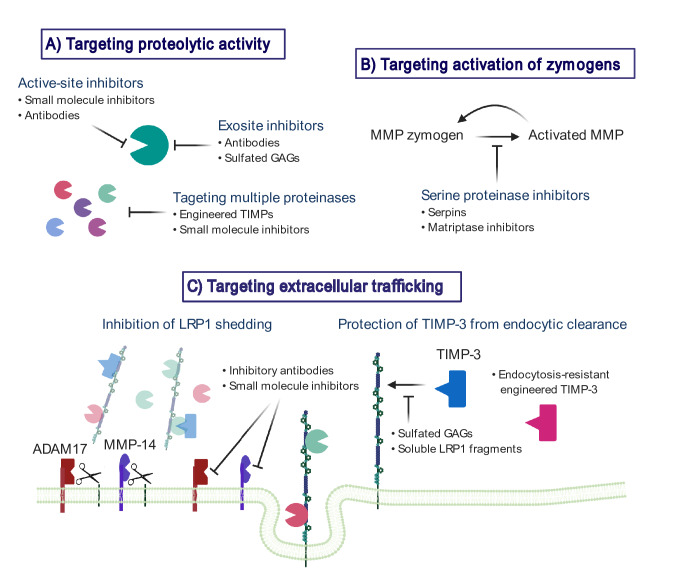
Targeting dysregulation of metalloproteinase activities at the extracellular milieu to protect cartilage. Metalloproteinases play essential roles under physiological conditions, inhibition of activities of metalloproteinases other than the target enzyme(s) most probably caused the side-effects. The agents that inhibit proteolytic activity of cartilage-degrading metalloproteinases (**a**) or activation of these enzymes (**b**) can be of benefit. On the other hand, since several metalloproteinases are involved in the degenerative processes, targeting multiple enzymes by engineered TIMPs (**a**), inhibition of LRP1 shedding (**c**) or prevention of TIMP-3 endocytosis (**c**) might be an effective way to protect cartilage from destruction

Considering that metalloproteinases play essential roles under both physiological and pathological conditions, inhibition of activities of metalloproteinases other than the target enzyme(s) likely results in side-effects. Indeed, the initial wave of MMP inhibitors offered poor selectivity and resulted in adverse effects such as musculoskeletal pain and tendonitis, and mild anaemia with elevated levels of liver enzymes. Increased understanding of the structure, function and regulation of individual metalloproteinases is thus critical for more effective strategies. On the other hand, since several metalloproteinases are involved in cartilage destruction, targeting multiple enzymes even with partial inhibition might be an effective way to protect cartilage from destruction (Fig. 2).

### Targeting Proteolytic Activity

#### Inhibitors Against Specific Metalloproteinases

In the human genome there are more than 50 closely related human metalloproteinases with similar basic active-site structures, which can make them susceptible to the same inhibitors. Among MMPs, MMP-13 is unusual as it has a very deep S1′ subsite. This feature has been exploited to generate highly selective MMP-13 inhibitors able to block collagen degradation in cartilage explants [110, 111] as well as animal OA models [112, 113] without musculoskeletal side-effects. Further evaluation of the therapeutic efficacy of these inhibitors is eagerly awaited.

The availability of highly specific ADAMTS-5 blocking antibodies [24, 114, 115] is a major breakthrough, since small molecule active-site inhibitors, despite the advantage of oral bioavailability, generally lack exquisite specificity and have broad side-effects, as noted above. Nevertheless, concerns about side-effects of ADAMTS-5 inhibitors are valid, mostly arising from observed roles of ADAMTS-5 in embryogenesis, as well as potentially in ECM turnover in the adult cardiovascular system [116–118]. A possible solution to bypassing a systemic toxicity is intra-articular administration of the blocking antibodies, but it has not been explored. Another challenge presented by OA is its long sub-clinical period, such that ADAMTS-5 inhibition may be most effective early in the disease process. Early treatment necessitates not only improvement of biomarkers for early OA diagnosis, but due consideration to long-term side-effects, or complications that may only become apparent decades after treatment initiation.

#### Exosite Inhibitors

Studies reporting an absolute requirement for the hemopexin domain of collagenases to cleave triple-helical collagens [119, 120], and the non-catalytic domains of ADAMTS-4 and 5 for cleaving aggrecan [21, 107, 108]. These studies suggest that the ancillary domains could be good targets for developing allosteric or exosite inhibitors that would exhibit higher specificity than conventional active site-directed inhibitors. Santamaria et al.[115] demonstrated that the antibody reacting with the spacer domain of ADAMTS-5 blocked the enzyme action only when aggrecan was substrates, but not against a peptide substrate. This finding supports the concept of exosite inhibitors for multidomain metalloproteinases.

#### Engineered TIMP-3 Against Multiple Metalloproteinases

The engineering of TIMPs has been an attractive approach to develop inhibitors for a group of metalloproteinases. The crystal structures of the TIMP-MMP complexes [121, 122] have provided clues to enable the modification of TIMPs towards greater selectivity. TIMP molecules have an extended ridge-like metalloproteinase interaction site. This region slots into the active site of metalloproteinases such that the amino and carbonyl groups of the N-terminal Cys^1^ residue chelate the active site zinc of the enzyme. Mutation around this reactive ridge of the TIMPs alters their specificity, giving selectivity for particular metalloproteinases. Addition of an extra alanine to the N-terminal of TIMP-3 (named [-1A]TIMP-3) or Thr^2^Gly mutation interferes with the inhibition of MMPs by TIMP-3, but inhibitory activity for ADAMTS-4 and 5, and ADAM17 is retained [30, 123]. This modified activity is driven by conformational changes, in which the active site is tilted and the interaction of Phe^34^ of the inhibitor with MMPs is lost.[-1A]TIMP-3 overexpressed in mouse cartilage led to a significant protection of the articular cartilage in a murine surgical model of OA [124] and a naturally occurring OA model in the STR/ort mice [125]. The unusual property of the specific inhibitor of aggrecanases and ADAM17 might provide a clue for the generation of a new type of inhibitor for metalloproteinases.

### Targeting Activation of Zymogens

Serine proteinase activity is countered by a host of anti-proteinases, the largest family of which are the serpins (serine proteinase inhibitors). Serpins are a unique and ancient family of proteins with an elegant mode of inhibition. Target proteinases cleave an extended reactive-centre loop or ‘bait region’, after which the serpin undergoes a major conformational change, and renders a hyperstable and covalent proteinase:serpin complex [126]. Serpins have been shown to have protective functions in cartilage. In the inflammatory collagen-induced arthritis model, intraperitoneal SERPINA1 (alpha-1 anti-trypsin) administration led to joint preservation and reduced inflammatory load, and this serpin also blocked collagen release from cytokine-stimulated bovine nasal cartilage [68, 127, 128]. We have recently observed that several other serpins are able to protect against collagen loss in this model (D. Wilkinson, unpublished observations). While the principle role of serpins is likely to be the inhibition of serine proteinases, serpins may have protective roles outside this inhibition. SERPINE2 has been demonstrated to downregulate expression MMP-13 in IL-1-stimulated chondrocytes. This inhibitory effect is likely regulated through a pathway involving ERK 1/2, NF-kappaB and the downstream transcription AP-1 [129].

Serine proteinases in the synovial joint will have multiple sources, including both the cartilage and synovium. The identification of specific MMP activators in arthritic disease is a major challenge which will likely differ depending on whether pathology is driven by an invasive synovium or the pathological activity of chondrocytes. Nevertheless, identification of disease-specific MMP activators could lead to novel avenues for therapy. One good candidate might be matriptase, since administration of small molecule inhibitors against the proteinase provided protection from cartilage loss in a murine model of OA [71].

### Targeting Extracellular Trafficking

#### Inhibition of LRP1 Shedding

Both ADAM17 and MMP-14 are responsible for shedding LRP1 in human chondrocytes [45]. However, their protein levels were not significantly changed between healthy and OA cartilage, suggesting that the activation process of these enzymes is post-translational. Importantly, combination of inhibitory antibodies against ADAM17 and MMP-14 blocks LRP1 shedding, restores endocytic capacity and reduces the degradation of aggrecan and collagen in OA cartilage [45]. An increase in LRP1 shedding in local tissues under inflammatory or chronic pathologic conditions may alter the trafficking of cartilage-degrading enzymes and TIMP-3. MMP-1 and MMP-3, whose *K*_D,app_ values for binding to immobilized LRP1 are > 1 µM, were markedly increased in the medium of human chondrocytes when endocytosis was blocked by a LRP ligand antagonist, receptor-associate protein (RAP)(K. Yamamoto, unpublished result). They are transcriptionally modulated presumably by increased factors that are normally endocytosed but elevated in the presence of LRP1 antagonists. Mantuano et al.[130] have shown similar transcriptional regulation for pro-inflammatory mediators, TNFα, IL-6 and CCL2, in macrophages when their LRP1-mediated endocytosis was blocked by RAP. In articular cartilage, such changes appear to dysregulate normal turnover of ECM and cellular homeostasis, leading to slowly progressing chronic diseases such as OA.

LRP1 endocytoses not only aggrecanases and collagenases but also a number of other secreted metalloproteinases either directly or through making a complex with TIMPs [59, 131, 132]. Inhibition of elevated LRP1 sheddase activities in OA cartilage may be an effective way to prevent cartilage matrix degradation. However, the systematic inhibition of ADAM17 and MMP-14 may be problematic as these enzymes are biologically important in the release of growth factors and cell surface receptors in many cell types [133, 134]. Thus, local administration of inhibitory antibodies or small molecule inhibitors for ADAM17 and MMP-14 may be worth investigating as disease-modifying OA drugs.

#### Protection of TIMP-3 from Endocytic Clearance

A canonical mode of ligand recognition by LRP1 underlies a central role for the cysteine-rich complement-type repeats (CRs). These cage-shaped CRs present a characteristic acidic pocket that forms salt bridges with a specific lysine on the ligand moiety, as it was shown for a number of LRP1 ligands [135, 136]. Previous structural and mutagenesis studies on LRP1 ligands have revealed that a proximal pair of lysine residues provides the dominant ligand-binding contribution to LRP1 [137, 138]. Doherty et al. [139] engineered LRP1-resistant mutants of TIMP-3 (Lys^26^Ala/Lys^45^Ala and Lys^45^Ala/Lys^110^Ala) without altering inhibitory activity against metalloproteinases. These TIMP-3 mutants have a longer half-life in cartilage and inhibit cartilage degradation at lower concentrations and for longer than wild-type TIMP-3. This illustrates that targeting the TIMP-3 endocytosis pathway is a potential strategy for inhibiting cartilage loss in OA. As mentioned above, sulphated GAGs including heparin, heparan sulphate and pentosan polysulphate are also able to inhibit TIMP-3 binding to LRP1, increase extracellular levels of TIMP-3 and thus protect cartilage ECM from degradation [60, 96]. However, such sulphated GAGs have poor pharmacokinetics and limited clinical scope. Chanalaris et al.[140] found that suramin, a polysulfphonated naphthalene derivative of urea, has similar chondroprotective activity to the sulphated GAGs. Although suramin’s clinical use has been limited by its adrenal toxicity, suramin might serve a scaffold for the development of novel therapeutics to target cartilage loss in OA.

The ligand-binding regions in LRP1 occur in four clusters (clusters I-IV) containing between 2 and 11 individual ligand-binding CRs. Most of the ligands for LRP1 for which the binding sites have been mapped interact with ligand-binding repeats in clusters II and IV [141]. As mentioned above, soluble LRP1 works as a decoy receptor and each soluble cluster binds to certain LRP1 ligands and inhibits endocytosis. Scilabra et al.[101] generated soluble mini-receptors (sLRPs) containing the four distinct binding clusters or part of each cluster. Interestingly, a soluble mini-receptor containing the N-terminal half of cluster II selectively blocked TIMP-3 internalisation, without affecting the turnover of ADAMTS-4, 5 or MMP-13. This soluble mini-receptor represents a biological tool that can be used to modulate TIMP-3 levels in the tissue. Engineering LRP1 clusters is thus a unique way to prevent endocytosis of certain LRP1 ligands in a selective manner.

## Conclusion and Perspective

OA is now widely accepted as a whole joint disease, and indeed all the joint structures including the adjacent bone surfaces, the synovial lining of the joint cavity, tendons, ligaments, and menisci are affected in OA. Although chondrocytes have been considered to be responsible for maintaining cartilage homeostasis by balancing synthesis and degradation of matrix molecules, it is still not clear that the loss of chondrocytes affects cartilage integrity or not [142]. Furthermore, to date, the role of proteinases in cartilage structural changes has been studied extensively, but their roles in synovial hypertrophy, osteophyte formation and subchondral bone remodelling is less well understood. Interestingly, [-1A]TIMP-3 overexpression led to a significant increase in bone mass, providing a novel concept that balance in aggrecanase and collagenase activities is crucial in bone remodelling [125].

Endocytic processes, including LRP1-mediated endocytosis, represent important mechanisms for regulating metalloproteinase activity, modulating extracellular levels of the enzymes and their endogenous inhibitors. In addition to endocytic scavenger functions, LRP1 can act as a signalling receptor via interaction of its cytoplasmic domain with various scaffolding and signalling proteins. A study by the Gonias group indicates that LRP1 can initiate different signalling pathways in response to binding of particular ligands to its extracellular domain [130]. This raises the possibility that LRP1-mediated uptake of metalloproteinases and their inhibitors is not merely a mechanism for clearing them from the extracellular environment, but that it also serves to deliver information to cells about turnover of their surrounding environment.

During the development of OA, cartilage ECM is slowly and gradually degraded. A prolonged period where biological changes are continuously taking place in a whole joint is a major challenge presented by the disease. If the rate of ECM degradation is reduced by the agents that inhibit activity of cartilage-degrading metalloproteinases—activation of these enzymes or LRP1 shedding without affecting the synthesis of ECM—this can be of benefit. However, inhibition of specific or multiple proteinases may be only effective at specific stages in the disease process. Further insights into regulations of individual enzymes in the complex environment at multiple stages of the OA development may allow us to develop effective approaches to ameliorate global joint pathology.

## References

[1] Stocker W, Bode W (1995). Structural features of a superfamily of zinc-endopeptidases: the metzincins. Curr Opin Struct Biol.

[2] Nagase H, Visse R, Murphy G (2006). Structure and function of matrix metalloproteinases and TIMPs. Cardiovasc Res.

[3] Gross J, Lapiere CM (1962). Collagenolytic activity in amphibian tissues: a tissue culture assay. Proc Natl Acad Sci USA.

[4] Dufour A, Overall CM (2015) Subtracting matrix out of the equation: new key roles of matrix metalloproteinases in innate immunity and disease. In: Matrix metalloproteinase biology. Wiley, Hoboken, pp 131–152

[5] Reiss K, Saftig P (2009). The "a disintegrin and metalloprotease" (ADAM) family of sheddases: physiological and cellular functions. Semin Cell Dev Biol.

[6] Apte SS (2009). A disintegrin-like and metalloprotease (reprolysin-type) with thrombospondin type 1 motif (ADAMTS) superfamily: functions and mechanisms. J Biol Chem.

[7] Apte SS, Parks WC (2015). Metalloproteinases: a parade of functions in matrix biology and an outlook for the future. Matrix Biol.

[8] Edwards DR, Handsley MM, Pennington CJ (2008). The ADAM metalloproteinases. Mol Asp Med.

[9] Lu P, Takai K, Weaver VM, Werb Z (2011). Extracellular matrix degradation and remodeling in development and disease. Cold Spring Harb Perspect Biol.

[10] Gunja-Smith Z, Nagase H, Woessner JF (1989). Purification of the neutral proteoglycan-degrading metalloproteinase from human articular cartilage tissue and its identification as stromelysin matrix metalloproteinase-3. Biochem J.

[11] Fosang AJ, Neame PJ, Hardingham TE, Murphy G, Hamilton JA (1991). Cleavage of cartilage proteoglycan between G1 and G2 domains by stromelysins. J Biol Chem.

[12] Yamamoto KSS, Suneel SA (2020). Analysis of aggrecanase activity using neoepitope antibodies. ADAMTS proteases: methods and protocols.

[13] Struglics A, Larsson S, Pratta MA, Kumar S, Lark MW, Lohmander LS (2006). Human osteoarthritis synovial fluid and joint cartilage contain both aggrecanase- and matrix metalloproteinase-generated aggrecan fragments. Osteoarthr Cartil.

[14] Sandy JD, Flannery CR, Neame PJ, Lohmander LS (1992). The structure of aggrecan fragments in human synovial fluid. Evidence for the involvement in osteoarthritis of a novel proteinase which cleaves the Glu 373-Ala 374 bond of the interglobular domain. J Clin Investig.

[15] Tortorella MD, Burn TC, Pratta MA, Abbaszade I, Hollis JM, Liu R, Rosenfeld SA, Copeland RA, Decicco CP, Wynn R, Rockwell A, Yang F, Duke JL, Solomon K, George H, Bruckner R, Nagase H, Itoh Y, Ellis DM, Ross H, Wiswall BH, Murphy K, Hillman MC, Hollis GF, Newton RC, Magolda RL, Trzaskos JM, Arner EC (1999). Purification and cloning of aggrecanase-1: a member of the ADAMTS family of proteins. Science.

[16] Glasson SS, Askew R, Sheppard B, Carito B, Blanchet T, Ma HL, Flannery CR, Peluso D, Kanki K, Yang Z, Majumdar MK, Morris EA (2005). Deletion of active ADAMTS5 prevents cartilage degradation in a murine model of osteoarthritis. Nature.

[17] Stanton H, Rogerson FM, East CJ, Golub SB, Lawlor KE, Meeker CT, Little CB, Last K, Farmer PJ, Campbell IK, Fourie AM, Fosang AJ (2005). ADAMTS5 is the major aggrecanase in mouse cartilage in vivo and in vitro. Nature.

[18] Little CB, Meeker CT, Golub SB, Lawlor KE, Farmer PJ, Smith SM, Fosang AJ (2007). Blocking aggrecanase cleavage in the aggrecan interglobular domain abrogates cartilage erosion and promotes cartilage repair. J Clin Investig.

[19] Little CB, Mittaz L, Belluoccio D, Rogerson FM, Campbell IK, Meeker CT, Bateman JF, Pritchard MA, Fosang AJ (2005). ADAMTS-1-knockout mice do not exhibit abnormalities in aggrecan turnover in vitro or in vivo. Arthritis Rheum.

[20] Glasson SS, Askew R, Sheppard B, Carito BA, Blanchet T, Ma HL, Flannery CR, Kanki K, Wang E, Peluso D, Yang Z, Majumdar MK, Morris EA (2004). Characterization of and osteoarthritis susceptibility in ADAMTS-4-knockout mice. Arthritis Rheum.

[21] Gendron C, Kashiwagi M, Lim NH, Enghild JJ, Thogersen IB, Hughes C, Caterson B, Nagase H (2007). Proteolytic activities of human ADAMTS-5: comparative studies with ADAMTS-4. J Biol Chem.

[22] Naito S, Shiomi T, Okada A, Kimura T, Chijiiwa M, Fujita Y, Yatabe T, Komiya K, Enomoto H, Fujikawa K, Okada Y (2007). Expression of ADAMTS4 (aggrecanase-1) in human osteoarthritic cartilage. Pathol Int.

[23] Song RH, Tortorella MD, Malfait AM, Alston JT, Yang Z, Arner EC, Griggs DW (2007). Aggrecan degradation in human articular cartilage explants is mediated by both ADAMTS-4 and ADAMTS-5. Arthritis Rheum.

[24] Larkin J, Lohr TA, Elefante L, Shearin J, Matico R, Su JL, Xue Y, Liu F, Genell C, Miller RE, Tran PB, Malfait AM, Maier CC, Matheny CJ (2015). Translational development of an ADAMTS-5 antibody for osteoarthritis disease modification. Osteoarthr Cartil.

[25] Scavenius C, Poulsen EC, Thogersen IB, Roebuck M, Frosticke S, Bou-Gharios G, Yamamoto K, Deleuran B, Enghild JJ (2019). Matrix-degrading protease ADAMTS-5 cleaves inter-alpha-inhibitor and release active heavy chain 2 in synovial fluids from arthritic patients. J Biol Chem.

[26] Fell HB, Barratt ME, Welland H, Green R (1976). The capacity of pig articular cartilage in organ culture to regenerate after breakdown induced by complement-sufficient antiserum to pig erythrocytes. Calcif Tissue Res.

[27] Karsdal MA, Madsen SH, Christiansen C, Henriksen K, Fosang AJ, Sondergaard BC (2008). Cartilage degradation is fully reversible in the presence of aggrecanase but not matrix metalloproteinase activity. Arthritis Res Ther.

[28] Troeberg L, Nagase H (2012). Proteases involved in cartilage matrix degradation in osteoarthritis. Biochim Biophys Acta.

[29] Pratta MA, Yao W, Decicco C, Tortorella MD, Liu RQ, Copeland RA, Magolda R, Newton RC, Trzaskos JM, Arner EC (2003). Aggrecan protects cartilage collagen from proteolytic cleavage. J Biol Chem.

[30] Lim NH, Kashiwagi M, Visse R, Jones J, Enghild JJ, Brew K, Nagase H (2010). Reactive-site mutants of N-TIMP-3 that selectively inhibit ADAMTS-4 and ADAMTS-5: biological and structural implications. Biochem J.

[31] Woolley DE, Glanville RW, Crossley MJ, Evanson JM (1975). Purification of rheumatoid synovial collagenase and its action on soluble and insoluble collagen. Eur J Biochem.

[32] Woolley DE, Crossley MJ, Evanson JM (1977). Collagenase at sites of cartilage erosion in the rheumatoid joint. Arthritis Rheum.

[33] Balbin M, Fueyo A, Knauper V, Lopez JM, Alvarez J, Sanchez LM, Quesada V, Bordallo J, Murphy G, Lopez-Otin C (2001). Identification and enzymatic characterization of two diverging murine counterparts of human interstitial collagenase (MMP-1) expressed at sites of embryo implantation. J Biol Chem.

[34] Cole AA, Chubinskaya S, Schumacher B, Huch K, Szabo G, Yao J, Mikecz K, Hasty KA, Kuettner KE (1996). Chondrocyte matrix metalloproteinase-8. Human articular chondrocytes express neutrophil collagenase. J Biol Chem.

[35] Hanemaaijer R, Sorsa T, Konttinen YT, Ding Y, Sutinen M, Visser H, van Hinsbergh VW, Helaakoski T, Kainulainen T, Ronka H, Tschesche H, Salo T (1997). Matrix metalloproteinase-8 is expressed in rheumatoid synovial fibroblasts and endothelial cells. Regulation by tumor necrosis factor-alpha and doxycycline. J Biol Chem.

[36] Yoshihara Y, Nakamura H, Obata K, Yamada H, Hayakawa T, Fujikawa K, Okada Y (2000). Matrix metalloproteinases and tissue inhibitors of metalloproteinases in synovial fluids from patients with rheumatoid arthritis or osteoarthritis. Ann Rheum Dis.

[37] Tchetverikov I, Lard LR, DeGroot J, Verzijl N, TeKoppele JM, Breedveld FC, Huizinga TW, Hanemaaijer R (2003). Matrix metalloproteinases-3, -8, -9 as markers of disease activity and joint damage progression in early rheumatoid arthritis. Ann Rheum Dis.

[38] Young DA, Barter MJ, Wilkinson DJ (2019) Recent advances in understanding the regulation of metalloproteinases. F1000Res 8 10.12688/f1000research.17471.1 PMC6381797 30828429

[39] Knauper V, Lopez-Otin C, Smith B, Knight G, Murphy G (1996). Biochemical characterization of human collagenase-3. J Biol Chem.

[40] Reboul P, Pelletier JP, Tardif G, Cloutier JM, Martel-Pelletier J (1996). The new collagenase, collagenase-3, is expressed and synthesized by human chondrocytes but not by synoviocytes. A role in osteoarthritis. J Clin Investig.

[41] Mitchell PG, Magna HA, Reeves LM, Lopresti-Morrow LL, Yocum SA, Rosner PJ, Geoghegan KF, Hambor JE (1996). Cloning, expression, and type II collagenolytic activity of matrix metalloproteinase-13 from human osteoarthritic cartilage. J Clin Investig.

[42] Little CB, Barai A, Burkhardt D, Smith SM, Fosang AJ, Werb Z, Shah M, Thompson EW (2009). Matrix metalloproteinase 13-deficient mice are resistant to osteoarthritic cartilage erosion but not chondrocyte hypertrophy or osteophyte development. Arthritis Rheum.

[43] Butler GS, Overall CM (2009). Updated biological roles for matrix metalloproteinases and new "intracellular" substrates revealed by degradomics. Biochemistry.

[44] Miller MC, Manning HB, Jain A, Troeberg L, Dudhia J, Essex D, Sandison A, Seiki M, Nanchahal J, Nagase H, Itoh Y (2009). Membrane type 1 matrix metalloproteinase is a crucial promoter of synovial invasion in human rheumatoid arthritis. Arthritis Rheum.

[45] Yamamoto K, Santamaria S, Botkjaer KA, Dudhia J, Troeberg L, Itoh Y, Murphy G, Nagase H (2017). Inhibition of shedding of low-density lipoprotein receptor-related protein 1 reverses cartilage matrix degradation in osteoarthritis. Arthritis Rheumatol.

[46] Kevorkian L, Young DA, Darrah C, Donell ST, Shepstone L, Porter S, Brockbank SM, Edwards DR, Parker AE, Clark IM (2004). Expression profiling of metalloproteinases and their inhibitors in cartilage. Arthritis Rheum.

[47] Bau B, Gebhard PM, Haag J, Knorr T, Bartnik E, Aigner T (2002). Relative messenger RNA expression profiling of collagenases and aggrecanases in human articular chondrocytes in vivo and in vitro. Arthritis Rheum.

[48] Holmbeck K, Bianco P, Caterina J, Yamada S, Kromer M, Kuznetsov SA, Mankani M, Robey PG, Poole AR, Pidoux I, Ward JM, Birkedal-Hansen H (1999). MT1-MMP-deficient mice develop dwarfism, osteopenia, arthritis, and connective tissue disease due to inadequate collagen turnover. Cell.

[49] Loeser RF, Goldring SR, Scanzello CR, Goldring MB (2012). Osteoarthritis: a disease of the joint as an organ. Arthritis Rheum.

[50] Mathiessen A, Slatkowsky-Christensen B, Kvien TK, Haugen IK, Berner Hammer H (2017). Ultrasound-detected osteophytes predict the development of radiographic and clinical features of hand osteoarthritis in the same finger joints 5 years later. RMD Open.

[51] Mathiessen A, Conaghan PG (2017). Synovitis in osteoarthritis: current understanding with therapeutic implications. Arthritis Res Ther.

[52] Bonewald LF (2011). The amazing osteocyte. J Bone Miner Res.

[53] Mazur CM, Woo JJ, Yee CS, Fields AJ, Acevedo C, Bailey KN, Kaya S, Fowler TW, Lotz JC, Dang A, Kuo AC, Vail TP, Alliston T (2019). Osteocyte dysfunction promotes osteoarthritis through MMP13-dependent suppression of subchondral bone homeostasis. Bone Res.

[54] Fanjul-Fernandez M, Folgueras AR, Cabrera S, Lopez-Otin C (2010). Matrix metalloproteinases: evolution, gene regulation and functional analysis in mouse models. Biochim Biophys Acta.

[55] Brew K, Nagase H (2010). The tissue inhibitors of metalloproteinases (TIMPs): an ancient family with structural and functional diversity. Biochim Biophys Acta.

[56] Yamamoto K, Murphy G, Troeberg L (2015). Extracellular regulation of metalloproteinases. Matrix Biol.

[57] Yamamoto K, Troeberg L, Scilabra SD, Pelosi M, Murphy CL, Strickland DK, Nagase H (2013). LRP-1-mediated endocytosis regulates extracellular activity of ADAMTS-5 in articular cartilage. FASEB J.

[58] Yamamoto K, Owen K, Parker AE, Scilabra SD, Dudhia J, Strickland DK, Troeberg L, Nagase H (2014). Low density lipoprotein receptor-related protein 1 (LRP1)-mediated endocytic clearance of a disintegrin and metalloproteinase with thrombospondin motifs-4 (ADAMTS-4): functional differences of non-catalytic domains of ADAMTS-4 and ADAMTS-5 in LRP1 binding. J Biol Chem.

[59] Yamamoto K, Okano H, Miyagawa W, Visse R, Shitomi Y, Santamaria S, Dudhia J, Troeberg L, Strickland DK, Hirohata S, Nagase H (2016). MMP-13 is constitutively produced in human chondrocytes and co-endocytosed with ADAMTS-5 and TIMP-3 by the endocytic receptor LRP1. Matrix Biol.

[60] Troeberg L, Fushimi K, Khokha R, Emonard H, Ghosh P, Nagase H (2008). Calcium pentosan polysulfate is a multifaceted exosite inhibitor of aggrecanases. FASEB J.

[61] Wilkinson DJ, Arques MDC, Huesa C, Rowan AD (2019). Serine proteinases in the turnover of the cartilage extracellular matrix in the joint: implications for therapeutics. Br J Pharmacol.

[62] Kappelhoff R, Puente XS, Wilson CH, Seth A, Lopez-Otin C, Overall CM (2017). Overview of transcriptomic analysis of all human proteases, non-proteolytic homologs and inhibitors: organ, tissue and ovarian cancer cell line expression profiling of the human protease degradome by the CLIP-CHIP DNA microarray. Biochim Biophys Acta Mol Cell Res.

[63] Blow DM, Birktoft JJ, Hartley BS (1969). Role of a buried acid group in the mechanism of action of chymotrypsin. Nature.

[64] Nagase H, Enghild JJ, Suzuki K, Salvesen G (1990). Stepwise activation mechanisms of the precursor of matrix metalloproteinase 3 (stromelysin) by proteinases and (4-aminophenyl)mercuric acetate. Biochemistry.

[65] Milner JM, Patel A, Davidson RK, Swingler TE, Desilets A, Young DA, Kelso EB, Donell ST, Cawston TE, Clark IM, Ferrell WR, Plevin R, Lockhart JC, Leduc R, Rowan AD (2010). Matriptase is a novel initiator of cartilage matrix degradation in osteoarthritis. Arthritis Rheum.

[66] Milner JM, Patel A, Rowan AD (2008). Emerging roles of serine proteinases in tissue turnover in arthritis. Arthritis Rheum.

[67] Caughey DE, Highton TC (1967). Components of the fibrinolytic system in synovial joints. Normal bovine compared with normal and abnormal human synovial joints. Ann Rheum Dis.

[68] Milner JM, Elliott SF, Cawston TE (2001). Activation of procollagenases is a key control point in cartilage collagen degradation: interaction of serine and metalloproteinase pathways. Arthritis Rheum.

[69] Hollander AP, Pidoux I, Reiner A, Rorabeck C, Bourne R, Poole AR (1995). Damage to type II collagen in aging and osteoarthritis starts at the articular surface, originates around chondrocytes, and extends into the cartilage with progressive degeneration. J Clin Investig.

[70] Knauper V, Will H, Lopez-Otin C, Smith B, Atkinson SJ, Stanton H, Hembry RM, Murphy G (1996). Cellular mechanisms for human procollagenase-3 (MMP-13) activation. Evidence that MT1-MMP (MMP-14) and gelatinase a (MMP-2) are able to generate active enzyme. J Biol Chem.

[71] Wilkinson DJ, Wang H, Habgood A, Lamb HK, Thompson P, Hawkins AR, Desilets A, Leduc R, Steinmetzer T, Hammami M, Lee MS, Craik CS, Watson S, Lin H, Milner JM, Rowan AD (2017). Matriptase induction of metalloproteinase-dependent aggrecanolysis in vitro and in vivo: promotion of osteoarthritic cartilage damage by multiple mechanisms. Arthritis Rheumatol.

[72] Falconer AMD, Chan CM, Gray J, Nagashima I, Holland RA, Shimizu H, Pickford AR, Rowan AD, Wilkinson DJ (2019). Collagenolytic matrix metalloproteinases antagonize proteinase-activated receptor-2 activation, providing insights into extracellular matrix turnover. J Biol Chem.

[73] Wilkinson DJ, Desilets A, Lin H, Charlton S, Del Carmen AM, Falconer A, Bullock C, Hsu YC, Birchall K, Hawkins A, Thompson P, Ferrell WR, Lockhart J, Plevin R, Zhang Y, Blain E, Lin SW, Leduc R, Milner JM, Rowan AD (2017). The serine proteinase hepsin is an activator of pro-matrix metalloproteinases: molecular mechanisms and implications for extracellular matrix turnover. Sci Rep.

[74] Gendron C, Kashiwagi M, Hughes C, Caterson B, Nagase H (2003). TIMP-3 inhibits aggrecanase-mediated glycosaminoglycan release from cartilage explants stimulated by catabolic factors. FEBS Lett.

[75] Black RA, Castner B, Slack J, Tocker J, Eisenman J, Jacobson E, Delaney J, Winters D, Hecht R, Bendele A (2006). A14 injected TIMP-3 protects cartilage in a rat meniscal tear model. Osteoarthr Cartil.

[76] Sahebjam S, Khokha R, Mort JS (2007). Increased collagen and aggrecan degradation with age in the joints of Timp3(−/−) mice. Arthritis Rheum.

[77] Mahmoodi M, Sahebjam S, Smookler D, Khokha R, Mort JS (2005). Lack of tissue inhibitor of metalloproteinases-3 results in an enhanced inflammatory response in antigen-induced arthritis. Am J Pathol.

[78] Javaheri B, Hopkinson M, Poulet B, Pollard AS, Shefelbine SJ, Chang YM, Francis-West P, Bou-Gharios G, Pitsillides AA (2016). Deficiency and also transgenic overexpression of Timp-3 both lead to compromised bone mass and architecture in vivo. PLoS ONE.

[79] Poulet B, Liu K, Plumb D, Vo P, Shah M, Staines K, Sampson A, Nakamura H, Nagase H, Carriero A, Shefelbine S, Pitsillides AA, Bou-Gharios G (2016). Overexpression of TIMP-3 in chondrocytes produces transient reduction in growth plate length but permanently reduces adult bone quality and quantity. PLoS ONE.

[80] Saw S, Aiken A, Fang H, McKee TD, Bregant S, Sanchez O, Chen Y, Weiss A, Dickson BC, Czarny B, Sinha A, Fosang A, Dive V, Waterhouse PD, Kislinger T, Khokha R (2019). Metalloprotease inhibitor TIMP proteins control FGF-2 bioavailability and regulate skeletal growth. J Cell Biol.

[81] Sawaji Y, Hynes J, Vincent T, Saklatvala J (2008). Fibroblast growth factor 2 inhibits induction of aggrecanase activity in human articular cartilage. Arthritis Rheum.

[82] Morris KJ, Cs-Szabo G, Cole AA (2010). Characterization of TIMP-3 in human articular talar cartilage. Connect Tissue Res.

[83] Milner JM, Rowan AD, Cawston TE, Young DA (2006). Metalloproteinase and inhibitor expression profiling of resorbing cartilage reveals pro-collagenase activation as a critical step for collagenolysis. Arthritis Res Ther.

[84] Chia SL, Sawaji Y, Burleigh A, McLean C, Inglis J, Saklatvala J, Vincent T (2009). Fibroblast growth factor 2 is an intrinsic chondroprotective agent that suppresses ADAMTS-5 and delays cartilage degradation in murine osteoarthritis. Arthritis Rheum.

[85] Lee HJ, Lee GH, Nah S, Lee KH, Yang H, Kim YM, Chun W, Hong S, Kim S (2008). Association of TIMP-4 gene polymorphism with the risk of osteoarthritis in the Korean population. Rheumatol Int.

[86] Herz J, Strickland DK (2001). LRP: a multifunctional scavenger and signaling receptor. J Clin Investig.

[87] Kowal RC, Herz J, Goldstein JL, Esser V, Brown MS (1989). Low density lipoprotein receptor-related protein mediates uptake of cholesteryl esters derived from apoprotein E-enriched lipoproteins. Proc Natl Acad Sci USA.

[88] Strickland DK, Ashcom JD, Williams S, Burgess WH, Migliorini M, Argraves WS (1990). Sequence identity between the alpha 2-macroglobulin receptor and low density lipoprotein receptor-related protein suggests that this molecule is a multifunctional receptor. J Biol Chem.

[89] Barrett AJ, Starkey PM (1973). The interaction of alpha 2-macroglobulin with proteinases. Characteristics and specificity of the reaction, and a hypothesis concerning its molecular mechanism. Biochemical J.

[90] Lillis AP, Van Duyn LB, Murphy-Ullrich JE, Strickland DK (2008). LDL receptor-related protein 1: unique tissue-specific functions revealed by selective gene knockout studies. Physiol Rev.

[91] Etique N, Verzeaux L, Dedieu S, Emonard H (2013). LRP-1: a checkpoint for the extracellular matrix proteolysis. Biomed Res Int.

[92] Herz J, Clouthier DE, Hammer RE (1992). LDL receptor-related protein internalizes and degrades uPA-PAI-1 complexes and is essential for embryo implantation. Cell.

[93] Omura TH, Noguchi A, Johanns CA, Jeffrey JJ, Partridge NC (1994). Identification of a specific receptor for interstitial collagenase on osteoblastic cells. J Biol Chem.

[94] Barmina OY, Walling HW, Fiacco GJ, Freije JM, Lopez-Otin C, Jeffrey JJ, Partridge NC (1999). Collagenase-3 binds to a specific receptor and requires the low density lipoprotein receptor-related protein for internalization. J Biol Chem.

[95] Troeberg L, Fushimi K, Scilabra SD, Nakamura H, Dive V, Thogersen IB, Enghild JJ, Nagase H (2009). The C-terminal domains of ADAMTS-4 and ADAMTS-5 promote association with N-TIMP-3. Matrix Biol.

[96] Scilabra SD, Troeberg L, Yamamoto K, Emonard H, Thogersen I, Enghild JJ, Strickland DK, Nagase H (2013). Differential regulation of extracellular tissue inhibitor of metalloproteinases-3 levels by cell membrane-bound and shed low density lipoprotein receptor-related protein 1. J Biol Chem.

[97] Nagase H, Kashiwagi M (2003). Aggrecanases and cartilage matrix degradation. Arthritis Res Ther.

[98] Fosang AJ, Rogerson FM (2010). Identifying the human aggrecanase. Osteoarthr Cartil.

[99] Walling HW, Raggatt LJ, Irvine DW, Barmina OY, Toledano JE, Goldring MB, Hruska KA, Adkisson HD, Burdge RE, Gatt CJ, Harwood DA, Partridge NC (2003). Impairment of the collagenase-3 endocytotic receptor system in cells from patients with osteoarthritis. Osteoarthr Cartil.

[100] Grimsley PG, Quinn KA, Owensby DA (1998). Soluble low-density lipoprotein receptor-related protein. Trends Cardiovasc Med.

[101] Scilabra SD, Yamamoto K, Pigoni M, Sakamoto K, Muller SA, Papadopoulou A, Lichtenthaler SF, Troeberg L, Nagase H, Kadomatsu K (2016). Dissecting the interaction between tissue inhibitor of metalloproteinases-3 (TIMP-3) and low density lipoprotein receptor-related protein-1 (LRP-1): development of a "TRAP" to increase levels of TIMP-3 in the tissue. Matrix Biol.

[102] Coveney CR, Collins I, Mc Fie M, Chanalaris A, Yamamoto K, Wann AKT (2018). Cilia protein IFT88 regulates extracellular protease activity by optimizing LRP-1-mediated endocytosis. FASEB J.

[103] Murphy G, Nagase H (2011). Localizing matrix metalloproteinase activities in the pericellular environment. FEBS J.

[104] Ra HJ, Parks WC (2007). Control of matrix metalloproteinase catalytic activity. Matrix Biol.

[105] Sakamoto S, Goldhaber P, Glimcher MJ (1973). Mouse bone collagenase. The effect of heparin on the amount of enzyme released in tissue culture and on the activity of the enzyme. Calcif Tissue Res.

[106] Yu WH, Woessner JF (2000). Heparan sulfate proteoglycans as extracellular docking molecules for matrilysin (matrix metalloproteinase 7). J Biol Chem.

[107] Kashiwagi M, Enghild JJ, Gendron C, Hughes C, Caterson B, Itoh Y, Nagase H (2004). Altered proteolytic activities of ADAMTS-4 expressed by C-terminal processing. J Biol Chem.

[108] Fushimi K, Troeberg L, Nakamura H, Lim NH, Nagase H (2008). Functional differences of the catalytic and non-catalytic domains in human ADAMTS-4 and ADAMTS-5 in aggrecanolytic activity. J Biol Chem.

[109] Troeberg L, Lazenbatt C, Anower EKMF, Freeman C, Federov O, Habuchi H, Habuchi O, Kimata K, Nagase H (2014). Sulfated glycosaminoglycans control the extracellular trafficking and the activity of the metalloprotease inhibitor TIMP-3. Chem Biol.

[110] Billinghurst RC, Dahlberg L, Ionescu M, Reiner A, Bourne R, Rorabeck C, Mitchell P, Hambor J, Diekmann O, Tschesche H, Chen J, Van Wart H, Poole AR (1997). Enhanced cleavage of type II collagen by collagenases in osteoarthritic articular cartilage. J Clin Investig.

[111] Piecha D, Weik J, Kheil H, Becher G, Timmermann A, Jaworski A, Burger M, Hofmann MW (2010). Novel selective MMP-13 inhibitors reduce collagen degradation in bovine articular and human osteoarthritis cartilage explants. Inflamm Res.

[112] Johnson AR, Pavlovsky AG, Ortwine DF, Prior F, Man CF, Bornemeier DA, Banotai CA, Mueller WT, McConnell P, Yan C, Baragi V, Lesch C, Roark WH, Wilson M, Datta K, Guzman R, Han HK, Dyer RD (2007). Discovery and characterization of a novel inhibitor of matrix metalloprotease-13 that reduces cartilage damage in vivo without joint fibroplasia side effects. J Biol Chem.

[113] Settle S, Vickery L, Nemirovskiy O, Vidmar T, Bendele A, Messing D, Ruminski P, Schnute M, Sunyer T (2010). Cartilage degradation biomarkers predict efficacy of a novel, highly selective matrix metalloproteinase 13 inhibitor in a dog model of osteoarthritis: confirmation by multivariate analysis that modulation of type II collagen and aggrecan degradation peptides parallels pathologic changes. Arthritis Rheum.

[114] Chiusaroli R, Visentini M, Galimberti C, Casseler C, Mennuni L, Covaceuszach S, Lanza M, Ugolini G, Caselli G, Rovati LC, Visintin M (2013). Targeting of ADAMTS5's ancillary domain with the recombinant mAb CRB0017 ameliorates disease progression in a spontaneous murine model of osteoarthritis. Osteoarthr Cartil.

[115] Santamaria S, Yamamoto K, Botkjaer K, Tape C, Dyson MR, McCafferty J, Murphy G, Nagase H (2015). Antibody-based exosite inhibitors of ADAMTS-5 (aggrecanase-2). Biochem J.

[116] Cikach FS, Koch CD, Mead TJ, Galatioto J, Willard BB, Emerton KB, Eagleton MJ, Blackstone EH, Ramirez F, Roselli EE, Apte SS (2018). Massive aggrecan and versican accumulation in thoracic aortic aneurysm and dissection. JCI Insight.

[117] Fava M, Barallobre-Barreiro J, Mayr U, Lu R, Didangelos A, Baig F, Lynch M, Catibog N, Joshi A, Barwari T, Yin X, Jahangiri M, Mayr M (2018). Role of ADAMTS-5 in aortic dilatation and extracellular matrix remodeling. Arterioscler Thromb Vasc Biol.

[118] Didangelos A, Mayr U, Monaco C, Mayr M (2012). Novel role of ADAMTS-5 protein in proteoglycan turnover and lipoprotein retention in atherosclerosis. J Biol Chem.

[119] Chung L, Dinakarpandian D, Yoshida N, Lauer-Fields JL, Fields GB, Visse R, Nagase H (2004). Collagenase unwinds triple-helical collagen prior to peptide bond hydrolysis. EMBO J.

[120] Manka SW, Carafoli F, Visse R, Bihan D, Raynal N, Farndale RW, Murphy G, Enghild JJ, Hohenester E, Nagase H (2012). Structural insights into triple-helical collagen cleavage by matrix metalloproteinase 1. Proc Natl Acad Sci USA.

[121] Gomis-Ruth FX, Maskos K, Betz M, Bergner A, Huber R, Suzuki K, Yoshida N, Nagase H, Brew K, Bourenkov GP, Bartunik H, Bode W (1997). Mechanism of inhibition of the human matrix metalloproteinase stromelysin-1 by TIMP-1. Nature.

[122] Fernandez-Catalan C, Bode W, Huber R, Turk D, Calvete JJ, Lichte A, Tschesche H, Maskos K (1998). Crystal structure of the complex formed by the membrane type 1-matrix metalloproteinase with the tissue inhibitor of metalloproteinases-2, the soluble progelatinase A receptor. EMBO J.

[123] Wei S, Kashiwagi M, Kota S, Xie Z, Nagase H, Brew K (2005). Reactive site mutations in tissue inhibitor of metalloproteinase-3 disrupt inhibition of matrix metalloproteinases but not tumor necrosis factor-alpha-converting enzyme. J Biol Chem.

[124] Nakamura H, Vo P, Kanakis I, Liu K, Bou-Gharios G (2006). Aggrecanase-selective tissue inhibitor of metalloproteinase-3 (TIMP3) protects articular cartilage in a surgical mouse model of osteoarthritis. Sci Rep.

[125] Kanakis I, Liu K, Poulet B, Javaheri B, van't Hof RJ, Pitsillides AA, Bou-Gharios G (2019). Targeted inhibition of aggrecanases prevents articular cartilage degradation and augments bone mass in the STR/Ort mouse model of spontaneous osteoarthritis. Arthritis Rheumatol.

[126] Huntington JA (2011). Serpin structure, function and dysfunction. J Thromb Haemost.

[127] Grimstein C, Choi YK, Wasserfall CH, Satoh M, Atkinson MA, Brantly ML, Campbell-Thompson M, Song S (2011). Alpha-1 antitrypsin protein and gene therapies decrease autoimmunity and delay arthritis development in mouse model. J Transl Med.

[128] Grimstein C, Choi YK, Satoh M, Lu Y, Wang X, Campbell-Thompson M, Song S (2010). Combination of alpha-1 antitrypsin and doxycycline suppresses collagen-induced arthritis. J Gene Med.

[129] Santoro A, Conde J, Scotece M, Abella V, Lois A, Lopez V, Pino J, Gomez R, Gomez-Reino JJ, Gualillo O (2015). SERPINE2 inhibits IL-1alpha-induced MMP-13 expression in human chondrocytes: involvement of ERK/NF-kappaB/AP-1 pathways. PLoS ONE.

[130] Mantuano E, Brifault C, Lam MS, Azmoon P, Gilder AS, Gonias SL (2016). LDL receptor-related protein-1 regulates NFkappaB and microRNA-155 in macrophages to control the inflammatory response. Proc Natl Acad Sci USA.

[131] Hahn-Dantona E, Ruiz JF, Bornstein P, Strickland DK (2001). The low density lipoprotein receptor-related protein modulates levels of matrix metalloproteinase 9 (MMP-9) by mediating its cellular catabolism. J Biol Chem.

[132] Bein K, Simons M (2000). Thrombospondin type 1 repeats interact with matrix metalloproteinase 2. Regulation of metalloproteinase activity. J Biol Chem.

[133] Hartmann M, Herrlich A, Herrlich P (2013). Who decides when to cleave an ectodomain?. Trends Biochem Sci.

[134] Itoh Y (2015). Membrane-type matrix metalloproteinases: their functions and regulations. Matrix Biol.

[135] van den Biggelaar M, Sellink E, Klein Gebbinck JW, Mertens K, Meijer AB (2011). A single lysine of the two-lysine recognition motif of the D3 domain of receptor-associated protein is sufficient to mediate endocytosis by low-density lipoprotein receptor-related protein. Int J Biochem Cell Biol.

[136] Strickland DK, Au DT, Cunfer P, Muratoglu SC (2014). Low-density lipoprotein receptor-related protein-1: role in the regulation of vascular integrity. Arterioscler Thromb Vasc Biol.

[137] Dolmer K, Campos A, Gettins PG (2013). Quantitative dissection of the binding contributions of ligand lysines of the receptor-associated protein (RAP) to the low density lipoprotein receptor-related protein (LRP1). J Biol Chem.

[138] Fisher C, Beglova N, Blacklow SC (2006). Structure of an LDLR-RAP complex reveals a general mode for ligand recognition by lipoprotein receptors. Mol Cell.

[139] Doherty CM, Visse R, Dinakarpandian D, Strickland DK, Nagase H, Troeberg L (2016). Engineered tissue inhibitor of metalloproteinases-3 variants resistant to endocytosis have prolonged chondroprotective activity. J Biol Chem.

[140] Chanalaris A, Doherty C, Marsden BD, Bambridge G, Wren SP, Nagase H, Troeberg L (2017). Suramin inhibits osteoarthritic cartilage degradation by increasing extracellular levels of chondroprotective tissue inhibitor of metalloproteinases 3. Mol Pharmacol.

[141] Neels JG, van Den Berg BM, Lookene A, Olivecrona G, Pannekoek H, van Zonneveld AJ (1999). The second and fourth cluster of class A cysteine-rich repeats of the low density lipoprotein receptor-related protein share ligand-binding properties. J Biol Chem.

[142] Zhang M, Mani SB, He Y, Hall AM, Xu L, Li Y, Zurakowski D, Jay GD, Warman ML (2016). Induced superficial chondrocyte death reduces catabolic cartilage damage in murine posttraumatic osteoarthritis. J Clin Invest.

